# GIGANTEA – an emerging story

**DOI:** 10.3389/fpls.2015.00008

**Published:** 2015-01-26

**Authors:** Priyanka Mishra, Kishore C. Panigrahi

**Affiliations:** Plant Science Lab, School of Biological Sciences, National Institute of Science Education and ResearchBhubaneshwar, India

**Keywords:** GIGANTEA, flowering time regulation, circadian clock control, GI mutants, light signaling

## Abstract

GIGANTEA (GI) is a plant specific nuclear protein and functions in diverse physiological processes such as flowering time regulation, light signaling, hypocotyl elongation, control of circadian rhythm, sucrose signaling, starch accumulation, chlorophyll accumulation, transpiration, herbicide tolerance, cold tolerance, drought tolerance, and *miRNA* processing. It has been five decades since its discovery but the biochemical function of GI and its different domains are still unclear. Although it is known that both *GI* transcript and GI protein are clock controlled, the regulation of its abundance and functions at the molecular level are still some of the unexplored areas of intensive research. Since GI has many important pleotropic functions as described above scattered through literature, it is worthwhile and about time to encapsulate the available information in a concise review. Therefore, in this review, we are making an attempt to summarize (i) the various interconnected roles that GI possibly plays in the fine-tuning of plant development, and (ii) the known mutations of *GI* that have been instrumental in understanding its role in distinct physiological processes.

## INTRODUCTION

**FIGURE 1:**
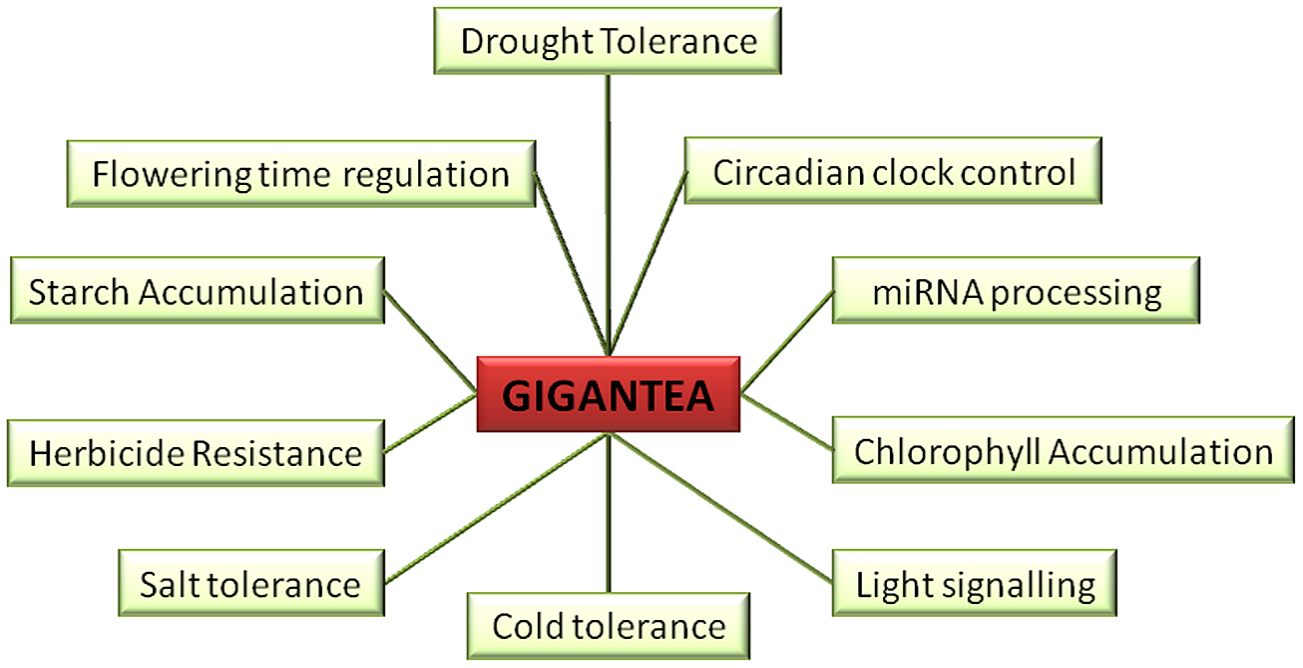
**Multiple roles of GIGANTEA (GI).** GI is known to play role in drought tolerance, circadian clock control, *miRNA* processing, chlorophyll accumulation, light signaling, cold tolerance, salt tolerance, herbicide resistance, starch accumulation, and flowering time regulation.

GIGANTEA (GI), the unique plant specific nuclear protein, although identified way back (Rédei, 1962) as a late flowering mutant *(gi)* in *Arabidopsis thaliana* (*At*), its precise biochemical roles are far from being understood (de Montaigu et al., 2010). The genomic organization of *GI* was evident after it was fine-mapped to chromosome 1 and subsequently, the *GI* cDNA was isolated (Fowler et al., 1999). The genomic locus of *GI* of *At* consists of 14 exons and encodes for a protein of 1173 amino acids (Fowler et al., 1999; Park et al., 1999). *GI* expression is ubiquitous and is detected throughout various stages of plant development indicative of its involvement in several functions summarized in **Figure 1** (Fowler et al., 1999; Park et al., 1999). It is interesting to note the ubiquitous expression of *GI* that reflect upon its pleiotropic roles in multitude of responses ranging from breaking of seed dormancy, hypocotyl elongation, initiating the circadian rhythm in seeds to the setting of fruits in the adult plant. Many of the above listed responses integrate information from the light input and external temperature, making it an interesting but complicated area of plant science.

Experiments aimed at understanding the abundance of the transcript and the protein are typically carried out in controlled cabinets, where the subjective time of the diurnal cycle are referred as the Zeitgeber time (ZT). Both the *GI* transcript and GI protein are under the control of diurnal regulation. Under long day (LD) growth cycle of 16 h light and 8 h dark (16 hL/8 hD), the *GI* mRNA peaks at ZT 10 and shows a trough at ZT 0, while under short day (SD) cycle of 8 hL/16 hD, *GI* transcript level peaks at ZT 8 (Fowler et al., 1999). The GI protein abundance also follows a similar pattern to its transcript accumulation (David et al., 2006). The regulation of GI is important for the control of circadian clock and several genes such as *FLAVIN-BINDING, KELCH REPEAT, F BOX 1* (*FKF1*), a blue light photoreceptor, and *CYCLING DOF FACTORs* (*CDFs*), which are involved in the transcription of a flowering time regulator *CONSTANS* (*CO*; Fornara et al., 2009). In addition, the diurnal regulation of the protein might also play an important role in the diurnal control of stomatal opening (Ando et al., 2013).

In order to assign a function to GI, it was of interest to enumerate its precise sub-cellular localization. Therefore, N-terminal GFP fusion of GI was constructed and transiently transfected in onion epidermal cells. The fluorescence microscopy of the fusion protein for the first time demonstrated that GI is predominantly localized to the nuclei and forms nuclear bodies (Huq et al., 2000). Later, the GI protein was also found to be localized in the nucleus of different cell types of transgenic *At* plants over-expressing *GI:GFP* (Mizoguchi et al., 2005). Four clusters of basic amino acids resembling the nuclear localization signal (NLS) in the GI sequence explained its nuclear abundance (Huq et al., 2000). GI has been shown to form nuclear bodies of diverse numbers, size, and shape (Kim et al., 2013c). To understand the molecular composition of GI nuclear bodies, attempts were made to evaluate the co-localization of GI with marker proteins of known sub-nuclear compartments such as heterochromatin bundles, nucleoli, spliceosome, and Cajal bodies. This piece of work demonstrated that GI did not localize to any of the above known nuclear compartments (Kim et al., 2013c). This suggested that GI might not have role in processes such as biogenesis of rRNA and snRNP, pre-mRNA splicing, and protein degradation. Since these co-localization studies were carried out in *Arabidopsis* mesophyll protoplasts using a transient over-expression method, it does not mimic the exact physiological environment. Furthermore, the association and dissociation rate of proteins to nuclear bodies has been shown to be affected by specific post-translational modifications. The spatio-temporal mis-localization of proteins can also affect its post-translational modifications. With so many complexities involved, stable transgenic lines expressing fluorescent tagged marker proteins and *GI* under their native promoters would be an impressive feat to achieve in order to understand the molecular composition of the GI complexes. Understanding the molecular composition of GI nuclear bodies (NBs) at different diurnal time-points would be a valuable asset.

The formation of GI nuclear bodies is light dependent since, the sequestration of GI into NBs is facilitated by EARLY FLOWERING 4 (ELF4) during the day, thus inhibiting the *CO* transcription. Likewise, EARLY FLOWERING 3 (ELF3) promotes the interaction of GI and CONSTITUTIVE PHOTOMORPHOGENESIS 1 (COP1) to form NBs which degrade GI in *planta* (Yu et al., 2008). The dynamic association of GI with heterogenous nuclear bodies during the light to dark transition needs to be evaluated. In other words, the question still remains, if GI associates and dissociates in a light dependent manner on a core complex within the nuclei based on its differential post-translational modification status.

Although studies showed the presence of GI predominantly in the nuclei, *in silico* analysis predicted the presence of 11 transmembrane domains in *At*GI which argues in favor of a possible membrane localization (Fowler et al., 1999). Furthermore, membrane localized GI possibly has a role in the regulation of ion channels during salt stress and stomatal opening as seen in phototropins (Stoelzle et al., 2003). Purified recombinant GI from *Escherichia coli* when subjected to electron microscopic study, revealed a tetrameric arrangement *in vitro*. However, its quaternary structure *in vivo* is still unclear (Black et al., 2011). This multimeric organization of a protein would not only offer more epitopes for interactions with diverse regulators but also would offer additional layers of control on its stability.

### ALLELES OF GIGANTEA WITH DISTINCT PHENOTYPES

**FIGURE 2:**
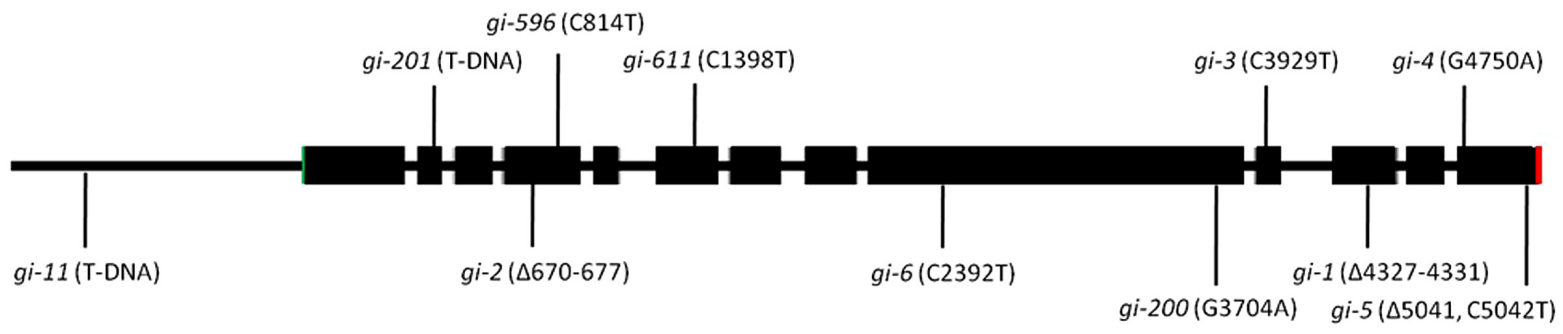
**The schematic presentation of the mutations in the *GI* locus.** The relative position of the available mutations in the genomic sequence of *GI* has been depicted. The start codon is indicated by green color while the stop position is represented by red. Coding regions are represented in black boxes while the non-coding regions are represented as lines.

The *gi* mutants were described as late flowering mutants for the first time (Rédei, 1962). There are several *gi* mutants described in literature such as *gi-1, gi-2, gi-3, gi-4, gi-5, gi-6, gi-11, gi-12, gi-100, gi-200, gi-201, gi-596,* and *gi-611* (summarized in **Table 1**; **Figure 2**). Some of the *gi* mutants were shown to influence the activity of the circadian clock, while others alter diverse responses (Park et al., 1999). The *gi-1* allele, lacking the C-terminal part of GI, was responsible for shortening the period of the clock, while the *gi-2* allele, lacking both the C-terminal and the central region of GI, lengthened the period. While the *gi-1* mutation shortened the period of *CAB*2 expression, the *gi-2* mutation lengthened the period of *CAB2* expression (Park et al., 1999). This suggests that the central region of the protein or the terminal half of the protein most probably fine-tunes the period length of the circadian clock.

**Table 1:** List of known *GI* alleles and their phenotypes.

No.	Allele name background site of mutation	Phenotypes							Key reference
		Flowering time	Starch content	Circadian rhythm	Herbicide tolerance	Hypocotyl length	Cold tolerance	Chlorophyll accumulation	
1	***gi-11*** Ws T-DNA insertion 407 bp upstream of start codon. 3–4 kb of the genomic sequence, removing the 5′ half of the gene and upstream sequence	Late^c^	NA	NA	NA	NA	NA	NA	TAIR; ^c^Fowler et al. (1999)
2	***gi-201*** Col-0 T-DNA insertion in second exon (66 amino acid, aa)	Late^f^	NA	Damped, altered circadian rhythm^f^	NA	Longer in RL and BL^f^	NA	NA	^f^Martin-Tryon et al. (2007)
3	***gi-2*** Col-0 Δ (670–677) bp premature stop codon (144 aa)	Late^a,c^	High^b^	Short; decreased period length^d^	NA	Longer in RL and BL^e,f^	NA	NA	TAIR; ^a^Araki and Komeda (1993), ^b^Eimert et al. (1995), ^c^Fowler et al. (1999), ^d^Park et al. (1999), ^e^Huq et al. (2000), ^f^Martin-Tryon et al. (2007)
4	***gi-596*** Ws S191F (C814T)	Not Affected ^j^	Long circadian period^j^	NA	NA	NA	NA	NA	^j^Gould et al. (2006)
5	***gi-611*** Ws L281F (C1398T)	Early in short photoperiod^j^	Short circadian period^j^	NA	NA	NA	NA	NA	TAIR; ^j^Gould et al. (2006)
6	***gi-6*** Ler-0 Q493Stop codon (C2392T) – 492 aa	Late^c^	NA	NA	NA	NA	NA	High^g^	TAIR; ^g^Kurepa et al. (1998a), ^c^Fowler et al. (1999)
7	***gi-200*** Col-0 S932N (G3704A)	NA	NA	Short circadian rhythm^f^	NA	Longer in RL and BL^f^	NA	NA	^f^Martin-Tryon et al. (2007)
		Flowering time	Starch content	Circadian rhythm	Herbicide tolerance	Hypocotyl length	Cold tolerance	Chlorophyll accumulation	
8	***gi-3*** Ler-0 Q964Stopcodon (C3929T) – 963 aa	Late^a,c,h^	High^b^	LHY, CCA1 expression lowered^h^	Tolerant toward paraquat, H_2_O_2_^g^	Longer in RL and BL^f,h^	Tolerant^i^	High^g^	TAIR; ^a^Araki and Komeda (1993), ^b^Eimert et al. (1995), ^g^Kurepa et al. (1998a), ^c^Fowler et al. (1999), ^i^Cao et al. (2005), ^h^Mizoguchi et al. (2005), ^f^Martin-Tryon et al. (2007)
9	***gi-1*** Col-0 Δ (4327–4331) bp	Late^a,c^	High^b^	Short; decreased period length^d^	NA	Longer in RL and BL^e,f^	NA	NA	TAIR; ^a^Araki and Komeda (1993), ^b^Eimert et al. (1995), ^c^Fowler et al. (1999), ^d^Park et al. (1999), ^e^Huq et al. (2000), ^f^Martin-Tryon et al. (2007)
10	***gi-4*** Ler-0 G4750A (G-splice acceptor site) – improper splicing. Premature stop codon (1083 aa).	Late^c^	NA	NA	NA	Longer in RL and BL^f^	Tolerant^i^	High^g^	TAIR; ^g^Kurepa et al. (1998a), ^c^Fowler et al. (1999), ^i^Cao et al. (2005), ^f^Martin-Tryon et al. (2007)
11	***gi-5*** Ler-0 C5042T and Δ5041 bp deletion	Late^c^	NA	NA	NA	NA	NA	High^g^	TAIR; ^g^Kurepa et al. (1998a), ^c^Fowler et al. (1999)
^∗^12	***gi-12*** Col-0 T-DNA insertion in GI coding region	Late^c^	NA	NA	NA	NA	NA	NA	^c^Fowler et al. (1999)
*13	***gi-100*** Col-0 3′ end unexpressed – ∼2 kb transcript produced	Late^e^	NA	NA	NA	Longer in RL^e^	NA	NA	^e^Huq et al. (2000)

Various reported alleles of *GI* based on the site of mutation and the corresponding phenotypes are depicted in the table below. In the case of *GI* alleles represented by *, the precise location of the mutations are not known and as such, arranged based on the time of discovery.

The *gi-2* mutant at higher temperature of about 28°C showed longer hypocotyl and flowered earlier in comparison to the plants grown at temperatures of 18 and 22°C (Araki and Komeda, 1993). Even though higher temperature were shown to regulate flowering (at 18, 22, 28°C) and hypocotyl elongation (at 22, 28°C) in *gi-2* mutant, it was almost equally sensitive toward vernalization as in WT. Vernalization is the exposure of plants to prolonged cold temperature that leads to earlier flowering cue in *Arabidopsis*. This implies that probably GI regulates flowering using a vernalization-independent pathway (Martinez-Zapater and Somerville, 1990; Koornneef et al., 1991; Araki and Komeda, 1993).

The alleles of *GI* are the result of random mutagenesis or T-DNA insertion which have aided in understanding its various functions. Alleles such as *gi-1*, *gi-2*, *gi-3,* and *gi-6* introduce premature stop codon whereas *gi-*4 and *gi-*5 most probably alter the C-terminus of the protein due to frame-shift mutations (Fowler et al., 1999). No *GI* expression was detected in the *gi-11* and *gi-201* alleles carrying T-DNA insertion (Richardson et al., 1998; Martin-Tryon et al., 2007). The *gi-100* mutation, originally identified in a red light screen, also contained a T-DNA insertion, but produced a truncated transcript of about 2 kb due to the absence of the 3′ end of *GI* (Huq et al., 2000). The transcript level in *gi-1*, *gi-2,* and *gi-3* is lower compared to that of *gi-4*, *gi-5, gi-*6, and *gi-100,* which show similar or higher levels compared to their respective WT (Fowler et al., 1999; Huq et al., 2000). The role of GI in blue light dependent hypocotyl elongation was revealed using the *gi-200* allele, consisting of a substitution of the serine 932 (Martin-Tryon et al., 2007).

Various deletions in *GI* sequences and its phenotypes are summarized in **Table 1**. After analyzing the data depicted in **Table 1**, it is evident that any deletion in *GI* mostly causes defects in the flowering time, circadian clock, and control of hypocotyl elongation. In the *gi-*4 mutant, improper splicing leads to a loss of 90 amino acids from the C-terminus causing late flowering. This deletion also causes the over-expression of its own transcript suggesting that the C-terminal 90 amino acids are required for its auto-regulation and flowering time (Fowler et al., 1999). The abundance of the *gi-*4 transcript could be due to increased stability or decreased decay which needs to be verified. Since GI stimulates *CO* transcription, this C-terminal domain of GI might be acting as an enhancer of *CO* transcription or involved in the recruitment of activators to the *CO* promoter.

The seeds of Wassilewskija (Ws) ecotype expressing *CAB:LUC* were mutagenized and screened for altered period length. Two novel alleles *gi-596* and *gi-611* were identified in this screen (Gould et al., 2006). In the *gi-596* allele, mutation caused by the substitution of the serine residue at 191 position to phenylalanine (S191F) did not affect the flowering-time although the period length of the circadian clock is lengthened and longer hypocotyl was observed under both red and blue light conditions. This suggests that the serine 191 residue might have an important role in photoreceptor signaling. On the contrary, the mutation in *gi-611* allele was mapped to the lysine 281. This allele showed significantly early flowering in SDs suggesting that this lysine in WT is involved in decelerating the flowering time (Gould et al., 2006). Since the Ws ecotype is a natural null for the high light sensor Phytochrome D, the phenotype observed could be a combinatorial effect of the lack of this photoreceptor and the respective mutations in *GI* allele (Aukerman et al., 1997). It would be interesting to evaluate if these alleles in Col background would show the similar light dependent effect to rule out the involvement and interaction of PHYD in this process. Both the positions, Lys281 and Ser191 are conserved in the Col-0 and Ler-0 ecotypes and thus, the role of these residues could be confirmed by the expression of the respective *GI* alleles containing the substitutions in these ecotypes to determine the importance of these mutated residues.

### TRANSCRIPTIONAL REGULATION OF *GIGANTEA*

Defects in the circadian clock components have been found to affect the *GI* transcription. CIRCADIAN CLOCK ASSOCIATED 1 (CCA1), a core component of the circadian clock, reduces the *GI* expression by binding to CCA1-binding site on *GI* promoter (Lu et al., 2012). *GI* transcript, thus accumulates toward the middle of the day, when *CCA1* expression is repressed by TIMING OF CAB EXPRESSION 1 (TOC1). The rhythmicity of *GI* transcript level is lost in *elf3* mutant in continuous light (LL) suggesting that ELF3 might also regulate the *GI* mRNA abundance (Fowler et al., 1999). Since CCA1 and ELF3 have been proposed to physically interact to control flowering time and hypocotyl elongation, it would be interesting to investigate the coordinated involvement of these two proteins in the regulation of *GI* transcription. Clock proteins, such as, LIGHT-REGULATED WD 1 and 2 (LWD1 and LWD2) also affect the *GI* expression pattern, since in *lwd1lwd2* double mutant *GI* transcript is most abundant at ZT 6 instead of ZT 10 (Wu et al., 2008). The two proteins being very similar (∼90% identity) possess functional redundancy, evident from single mutants being phenotypically similar to WT. Another clock associated gene, *TIME FOR COFFEE (TIC)* is also known to regulate the rhythmicity of *GI* in *Arabidopsis*. In *tic* mutants, *GI* transcript level is lower and the peak is shifted ∼4 h earlier than in WT plants (Hall et al., 2003). Since in both the *lwd1lwd2* and *tic* mutants the *GI* expression is shifted to ZT6, it suggests that the activities of both the proteins might be required for the repression of the *GI* transcription in the morning. pseudo-response regulators (PRRs), namely, PRR5, PRR7, and PRR9 also have been proposed to regulate *GI* expression and therefore, flowering time via the CO-FT module (Nakamichi et al., 2007; Kawamura et al., 2008). Epistatic analysis and mutant combinations between *LWD1/2, PRRs,* and *TIC* would be beneficial to explain the additive roles of these genes products and the genetic hierarchy of the genes regulating the inhibition of *GI* expression. The expression of *GI* at the wrong time of the diurnal cycle is known to cause flowering time defects in *At* (Fornara et al., 2009). These mutants might behave as late flowering due to the untimely expression of *GI*. Although a lot is known from the transcript analysis, the work at the protein level is far from being understood due to the unavailability of a GI anti-serum that could detect the endogenous GI protein. The detailed post-translational regulation of GI is explained in the Section “Post-Translational Regulation of GIGANTEA.”

Several studies have demonstrated that light quality and quantity influence *GI* transcription, although systematic studies involving changes in the light fluence and wavelength to evaluate *GI* expression is yet to be carried out. In *Arabidopsis*, upon transition to night, *GI* mRNA level decreases with a half-life of about 1 h irrespective of the photoperiod (Fowler et al., 1999). A significant light dependent down-regulation is also detected in the legume *Medicago truncatula* suggesting a similar mechanism might coordinate light sensing with transcriptional activity (Paltiel et al., 2006). *GI* mRNA accumulation pattern in both *Arabidopsis* and *M. truncatula* showed a secondary peak at ZT 2 under SDs as well as LDs (Paltiel et al., 2006). This peak could be the result of an acute response to light. A similar peak of *GI* mRNA at ZT 2 has also been documented in plants grown under blue light. The role of blue light in the regulation of this early secondary peak of *GI* needs to be thoroughly examined using mutants that are affected in blue light signaling. This would clarify if the peak at ZT 2 is due to photoreceptors or secondary signaling components involved in blue light signaling. The peak expression of *GI* is delayed by approximately 4 h in plants grown in low red:far-red (R:FR) light conditions in comparison to plants grown in white light condition (Wollenberg et al., 2008). This indicated that the photoreceptors and their activity are fine-tuning the timing and quantity of the GI transcript. The accumulation of the GI protein in the morning around ZT 3–4 and its consequence in plant development has not been studied yet, that needs to be evaluated in depth.

Besides light, temperature too has been found to regulate *GI* expression. Warmer temperature of 28°C up-regulates *GI* mRNA level as compared to the cooler temperatures of 12°C at dawn (Paltiel et al., 2006). The night time repression of *GI* transcription was shown to be temperature dependent and regulated by evening complex (EC) night time repressor constituted of ELF3, ELF4, and LUX ARRHYTHMO (LUX; Nusinow et al., 2011; Mizuno et al., 2014). The EC night time repressor was revealed to bind to the *GI* promoter through LUX binding site (LBS).

GIGANTEA has been proposed to regulate its own expression, since the mutants, *gi-1* and *gi-2,* have lower expression of the GI alleles, approximately 40 and 20% of the WT transcript, respectively (Fowler et al., 1999; Park et al., 1999). But this auto-regulatory role of *GI* transcription is contradictory, since, *gi-4* and *gi-6* lines show ∼30% higher expression of *GI* compared to its WT (Fowler et al., 1999). This effect could be either due to the difference in the ecotypes or differential regulation of the transcript stability. Another question worth investigating would be the abundance of the mutant proteins produced in each mutant, which would require a functional GI antiserum. The positive or negative auto-regulatory role suggests that mutations at different residues in the coding sequence can influence the abundance of transcriptional enhancers or repressors, affecting *GI* expression.

### POST-TRANSLATIONAL REGULATION OF GIGANTEA

Over-expression of *GI* leads to the constitutive accumulation of the *GI* transcript throughout the photoperiod. Despite its constant expression level, GI protein follows a cyclic pattern of accumulation in both LDs and SDs. This is suggestive of the degradation of the GI protein (David et al., 2006). GI was found to be ubiquitinated upon dusk, a pre-requisite for its degradation via the 26S proteasome mechanism (David et al., 2006). In the dark phase, nuclear GI abundance has been shown to be regulated by the E3 ubiquitin ligase activity of COP1 and ELF3 (Yu et al., 2008). The interaction between COP1 and GI is ELF3 dependent, where ELF3 serves as an adaptor protein (Yu et al., 2008). The shuttling of COP1 between the nucleus and the cytoplasm is regulated by light (von Arnim and Deng, 1994). COP1 being nuclear localized in the night phase makes it competent for COP1–ELF3 mediated degradation of GI through 26S proteasome.

**FIGURE 3:**
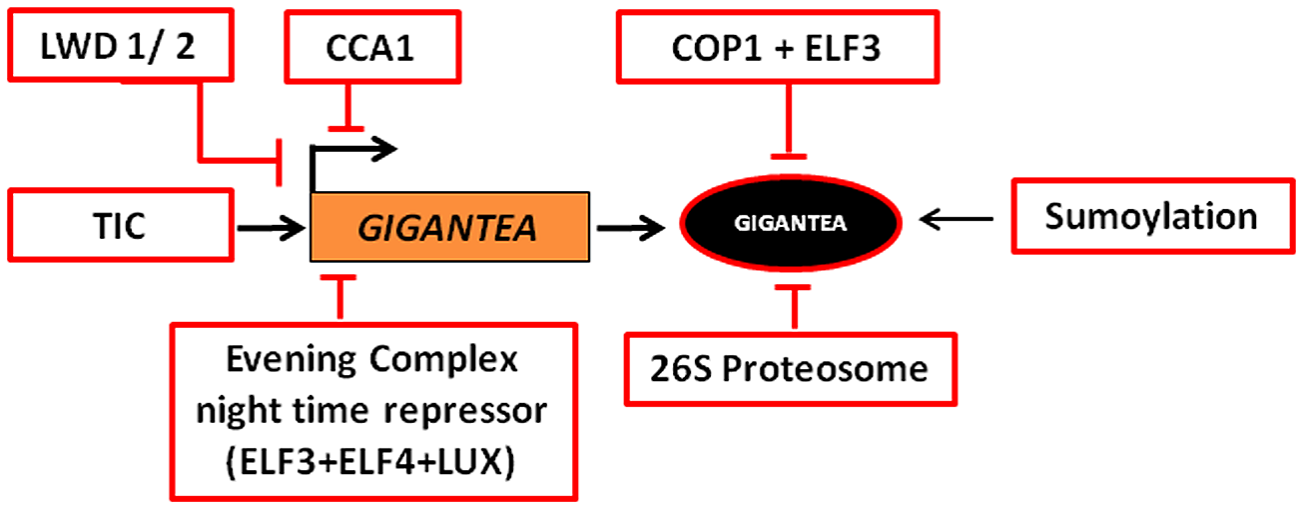
**Transcriptional and post-translational regulation of GI.** The expression of *GI* is regulated by LIGHT-REGULATED WD1/2 (LWD1/2), TIME FOR COFFEE (TIC), and CIRCADIAN CLOCK ASSOCIATED 1 (CCA1) in the day and EVENING COMPLEX (EC) night time repressor during night, such that the transcript peaks at around ZT 10 in LD and ZT 8 in SD. In the dark phase, 26S Proteasome, CONSTITUTIVE PHOTOMORPHOGENESIS 1 (COP1), and EARLY FLOWERING 3 (ELF3) regulate the abundance of the protein. SUMOylation of GI stabilizes the protein.

Upon heat shock GI is SUMOylated (López-Torrejón et al., 2013). It has been proposed that SUMOylation prevents the degradation of GI, thus enhancing its abundance. GI accumulation has been correlated with earlier flowering under heat stress. The identification of SUMOylation and ubiquitination sites in GI that alter its stability and degradation could be of pivotal importance in manipulating flowering time of crop plants. Current knowledge on the transcriptional and post-translational regulation of GI is presented schematically in **Figure 3**.

## ROLES OF GIGANTEA

GIGANTEA plays multiple roles throughout plant development. Its functions in processes such as light signaling, circadian clock regulation, flowering time control, chlorophyll accumulation, sugar metabolism, and stress tolerance have been discussed below.

### LIGHT SIGNALING

Photoreceptors such as phytochromes, cryptochromes, UV-light receptor, and phototropins help plants to sense variations in the light quality, quantity, and direction. The red and far-red light photoreceptors, phytochromes, are encoded by a multigene family, PhyA–E in *Arabidopsis*. While PhyA is the far-red light receptor, PhyB–E function as red light receptors with PhyB playing a predominant role. They mediate very-low-fluence responses (VLFRs), low-fluence responses (LFRs), and the high-irradiance responses (HIRs), with reference to the photon flux density (Casal et al., 1998). Like *phyB-9* mutant, *gi-100* also shows elongated hypocotyl when grown under saturated red light (Huq et al., 2000). Neither the genes nor the proteins abundance of PhyA and PhyB are influenced in *gi-100* (Huq et al., 2000). Therefore, GI was suggested to function downstream of PhyA and PhyB. Mutation in *GI* leads to decreased VLFR under FR light suggesting its role in PhyA signaling (Oliverio et al., 2007). The *gi* mutants showed reduced seed germination and cotyledon unfolding in FR light conditions. These phenotypes are rescued by over-expression of *GI.* This suggested that GI might have a positive role specifically in PhyA mediated VLFR. GI also has a role in regulating flowering in low R:FR ratio which might be attributed to PhyA signaling (Wollenberg et al., 2008). Both PhyA and PhyB form NBs like GI. It would be interesting to determine if Phys and GI are present in the same sub-nuclear complexes and the localization of GI in the NBs alters the Phy-mediated functions.

The *gi* mutants showed longer hypocotyl in comparison to WT under blue light (Martin-Tryon et al., 2007). Earlier, it had been suggested that GI may be either a positive regulator of TOC1 or act parallel to it for the regulation of hypocotyl elongation. Since only *gi* not *toc1* mutants show the longer hypocotyl in blue light, it can be inferred that GI does not regulate TOC1 for hypocotyl elongation (Martin-Tryon et al., 2007).

### CIRCADIAN CLOCK CONTROL

The circadian clock controls many processes depending on the length of the day and night cycle in an organism. In plants, the rhythmic expressions of various genes are influenced by the circadian clock, thereby regulating functions such as elongation of hypocotyl, petioles and inflorescence stem, movement of cotyledon and leaf, and flowering time. CCA1, LATE ELONGATED HYPOCOTYL (LHY), and TOC1 are the core components of circadian oscillator in plants (Somers, 1999). In 2005, the clock was proposed to be an interlocking network of proteins working in a feedback loop (Locke et al., 2005). According to the new model of clock, while the morning elements LHY and CCA1 repress *TOC1* transcription, the evening element TOC1 down-regulates *LHY/CCA1* accumulation, differing with the earlier observations (Alabadí et al., 2001; Gendron et al., 2012; Huang et al., 2012).

To understand the circadian rhythm in *Arabidopsis*, the ESPRESSO Quantitative Trait Loci (QTL) was generated from the cross between Ler and Cvi ecotypes (Swarup et al., 1999). Ler and Cvi ecotypes were suggested to comprise of an even distribution of alleles involved in the shortening and lengthening of period, since the progeny of their cross generated lines which had period length both longer and shorter than the parents. *GI* was identified as one of the genes that could be responsible for regulating the rhythms of cotyledon movement (Park et al., 1999; Swarup et al., 1999). The *gi* mutants have diverse circadian periods than WT concluding that GI has a role in period length regulation. Mutation in *GI* affects *CHLOROPHYLL A/B-BINDING PROTEIN 2* (*CAB2*) gene expression which is also under the control of circadian clock (Park et al., 1999).

Soon after a day of imbibition of seeds, GI is required for initiating the rhythmicity of the circadian clock (Salomé et al., 2008). Mutations in the *GI* locus affect the *CCA1* and *LHY* gene expression in both LDs and SDs conditions (Fowler et al., 1999). A recent study proposed that both the nuclear and cytosolic GI are required to positively and negatively regulate *LHY* expression, respectively, that fine-tunes the clock function (Kim et al., 2013b). Over-expression or mutations of *CCA1* and *LHY* disrupted the *GI* expression (Fowler et al., 1999). Accordingly, the double mutant of *LHY* and *CCA1* showed early abundance of *GI* transcript (Mizoguchi et al., 2002, 2005). It suggests that GI operates in a feedback loop as a component to maintain the rhythmicity and period length of the clock.

The established LHY/CCA1-TOC1 module of the clock could not explain the experimental data like the time difference of about 12 h between *LHY/CCA1* abundance in morning and *TOC1* accumulation in evening (Alabadí et al., 2001; Locke et al., 2005). It was therefore proposed that LHY/CCA1-TOC1 module comprises of other components. One of the components was predicted to be *GI*, whose expression followed the same pattern as predicted by the *in silico* analysis and was subsequently experimentally confirmed (Locke et al., 2006). Further work suggested that GI alone would not be able to regulate the observed time difference (Kawamura et al., 2008). TOC1 in turn is regulated by GI along with ZEITLUPE (ZTL), an F-box protein (Kim et al., 2011). ZTL is a blue light photoreceptor which is stabilized by its interaction with GI and Heat Shock Protein 90 (HSP90). Together the ZTL-GI complex control TOC1 level (Kim et al., 2007).

Temperature compensation is an important characteristic of the circadian clock to maintain the rhythm over a range of environmental temperature. GI was recognized as a candidate regulating temperature compensation effect, especially at higher temperatures (Edwards et al., 2005; Gould et al., 2006). Since fluctuations in the temperature regulate the abundance of *GI* transcript, it could be plausible that *GI* and temperature sensing mechanism crosstalk and feedback each other.

**FIGURE 4:**
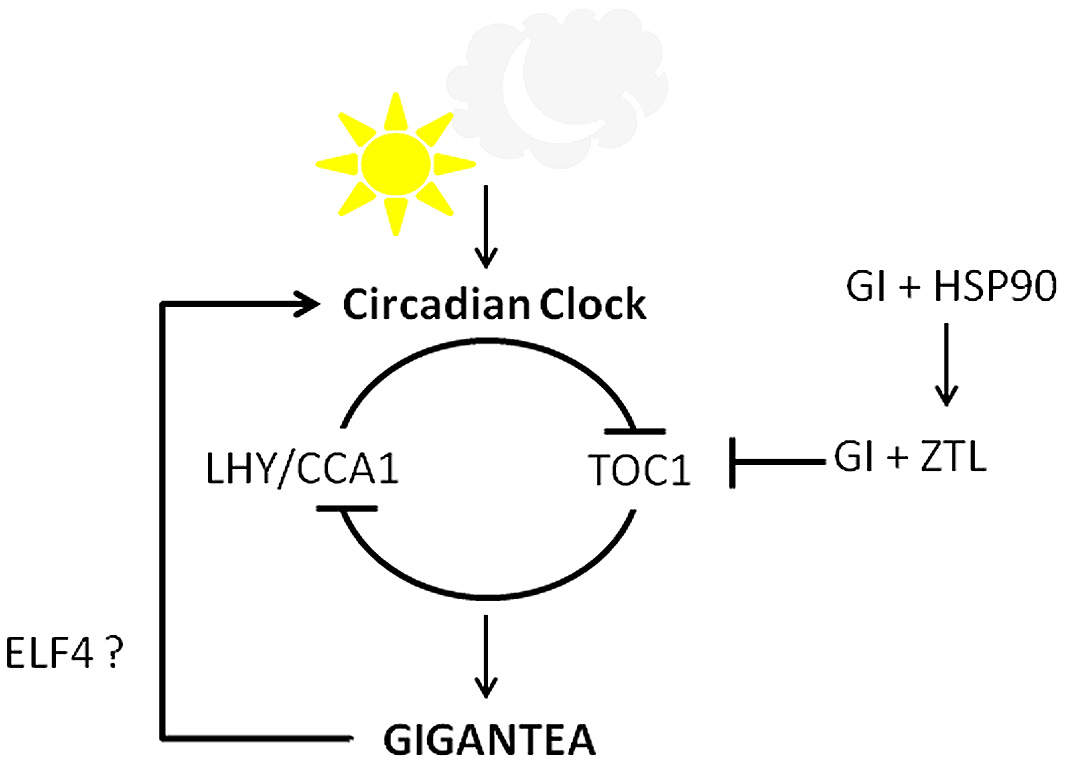
**Circadian clock control by GI.** GI and the central clock components work in a feedback loop. GI along with ELF4 positively regulates the clock while GI and ZTL form a complex to degrade TOC1 in evening. GI and HSP90 regulate the stability of ZTL, thereby influencing clock.

*Arabidopsis thaliana* dawn and dusk, GI regulates the clock rhythm along with ELF4 (Kim et al., 2012). GI was also required for iron-deficiency induced long circadian clock rhythm (Chen et al., 2013). Reduced depolymerization of actin filament caused the period of the circadian clock to shorten, as evident from the shortened period of *GI* expression (Tóth et al., 2012). Since *GI* expression is under the control of the circadian clock, *GI* accumulation pattern has been exploited to screen for novel clock mutants (Onai et al., 2004). Many components that mediate between GI and the clock are still to be unraveled. The role of GI in the regulation of the clock documented till date is summarized in **Figure 4**.

### PHOTOPERIODIC FLOWERING-TIME REGULATION

GIGANTEA is a major mediator between the circadian clock and the master regulator of photoperiodic flowering time control, CO. GI upregulates *CO* transcription, thereby accelerating time required to flower. Koornneef et al. (1998) showed that a novel mutant, *gi-3,* is epistatic to *CO* and *FLOWERING LOCUS T (FT)* way back. Mutation in *GI* led to a decrease in the accumulation of *CO* mRNA without affecting its cycling phase compared to its WT that led to delayed flowering (Suárez-López et al., 2001). Mutants in the *GI* locus or over-expressors of *GI* did not discriminate day-length for flowering. Accordingly, *gi* mutants were later flowering and over-expressors were earlier in both LDs and SDs (Rédei, 1962; Araki and Komeda, 1993; Mizoguchi et al., 2005).

**FIGURE 5:**
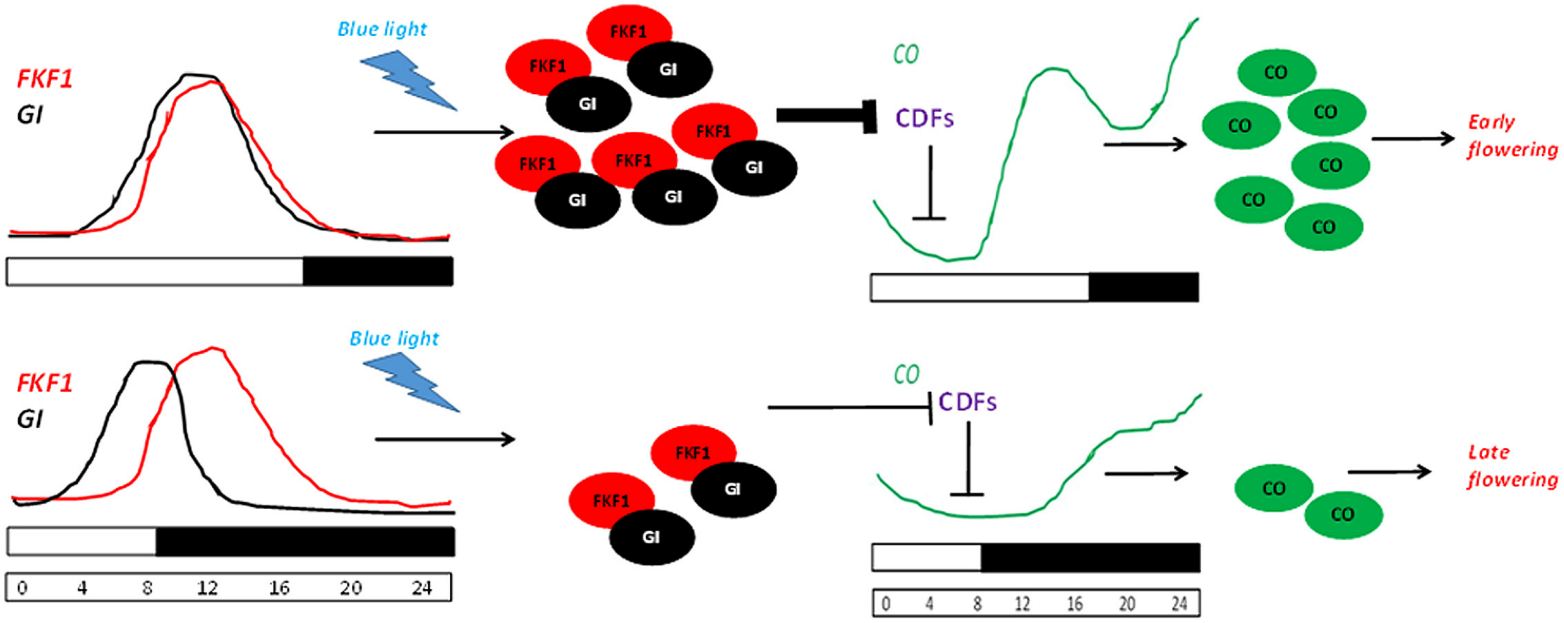
**GI-FKF1 complex regulates the photoperiodic flowering time.** In LD, the peak expression of *GI* and *FLAVIN-BINDING KELCH REPEAT F-BOX 1 (FKF1)* coincide, leading to the accumulation of GI-FKF1 complex. The GI-FKF1 complex regulates the degradation of Cycling DOF factors (CDFs) bound to the *CONSTANS (CO)* promoter. In the absence of the inhibitor *CO* transcription occurs resulting in the accumulation of CO protein that promotes flowering. While in SDs, less abundance of GI-FKF1 complex does not degrade CDFs repressing *CO* transcription.

During dawn, *CO* transcription is repressed by the combinatorial activity of the DOF transcription repressors bound to the *CO* promoter. In LDs, the expression of *FKF1* and *GI* coincide at ZT10. Therefore, toward the middle of the day the accumulation of GI along with FKF1 forms a complex competent to degrade the DOF factors. This elevates the *CO* transcription, thereby leading to *FT* expression (Imaizumi et al., 2003, 2005; Sawa et al., 2007). While in SDs, since FKF1 accumulates 3 h after GI peaks, it does not allow the formation of the degradation complex, therefore leading to a low abundance of *CO* transcript. This photoperiod pathway where GI regulates *FT* expression in a CO-dependent manner is schematically depicted in **Figure 5**. GI regulates the abundance of FKF1, which is involved in the proteasomal degradation of proteins (Fornara et al., 2009). Post-transcriptionally, GI also controls the sub-cellular level of *CYCLING DOF FACTOR 2* (*CDF2*; Fornara et al., 2009). FKF1 belongs to a family of F-Box proteins containing two other candidates – LOV KELCH Protein 2 (LKP2) and ZTL. The blue light dependent interaction between GI and FKF1 is mediated by the LOV (Light, Oxygen, or Voltage) domain of FKF1 and the amino-terminal of GI *in vivo* (Sawa et al., 2007). The *gi-100* mutant is later flowering than the F-Box triple mutant *fkf1 ztl-4 lkp2-1*. This might be due to the presence of GI in *fkf1 ztl-4 lkp2-1*, which down-regulates the abundance of *CDF* transcripts, or the presence of an additional layer of control by GI bypassing the triple F-Box module.

There are at least two independent mechanisms through which GI regulates *FT* expression independent of CO. While the first mechanism involves microRNA, the second mechanism is through the binding of GI to the *FT* promoter. The microRNA based control involves *miRNA172,* which is positively regulated in the presence of GI. The *miR172* inhibits the expression of *TARGET OF EAT1* (*TOE1*), an APETALA 2 (AP2)-related transcriptional repressor of *FT* (Jung et al., 2007). In the recent past, expression of *GI* specifically in the mesophyll or vascular tissue was carried out. This rescued the late-flowering phenotype of *gi-2* under both day length conditions and two different temperatures of 16 and 23°C (Sawa and Kay, 2011). The expression of *GI* in mesophyll and vascular tissue was done using tissue specific promoters *LIGHT-HARVESTING COMPLEX B2.1* (*pLhCB2.1*) and *SUCROSE TRANSPORTER 2* (*pSUC2*), respectively. While expression pattern of *GI* under the control of *pLhCB2.1* is altered and peaked at ZT 0, *GI* expressed under the phloem specific promoter led to the over-expression of the transcript with peak at ZT 10. The *FT* transcript level was up-regulated without an increase in *CO* mRNA in both day-length conditions. GI was shown to binds to the *FT* transcriptional repressors such as SHORT VEGETATIVE PHASE (SVP), TEMPRANILLO 1 (TEM1), and TEMPRANILLO 2 (TEM2), and their specific target regions within the *FT* promoter in the mesophyll, thereby relieving the repression and promoting *FT* transcription (Sawa and Kay, 2011). The degradation of the *FT* transcriptional repressors or the unavailability of their binding sites on the *FT* promoters due to the presence of GI could lead to the abundance of the *FT* transcript. *FT* expressed in the vascular tissue normally triggers flowering. Since *GI* expressed in mesophyll accelerated flowering, elevating the FT level in vasculature, the signal from mesophyll GI most likely induces *CO* transcription in vasculature. Alternatively, the GI could be transported to the vascular tissues and induce the photoperiod module which needs to be investigated.

Expression of *35S::GI:GFP* in *gi-3* plants complemented the late flowering phenotype of *gi-3.* On the contrary, expressing the *35S::GFP:GI* in *gi-3* caused later flowering compared to the background lines indicating that the N-terminal fusion of GI might be either non-functional or might not be imported into the nucleus. In the transgenic line expressing C-terminal fusion, the fusion protein was localized to the nucleus and formed NBs (Mizoguchi et al., 2005). In an independent study, transgenic plants expressing glucocorticoid receptor (GR) fusion of GI flowered with ∼20 leaves less when treated with dexamethasone, compared to its untreated control which flowered with ∼55 leaves under LDs (Günl et al., 2009). In 15 day old seedlings, the induction of flowering time genes like *CO* and *FT* took place ∼28 h after dexamethasone treatment causing early flowering. This indicates that cytoplasmic retention of GI most probably delays time to flower. Mutation in *GI* is epistatic to mutation in *ELF4* and together regulate *CO* expression (Kim et al., 2012). Recent studies showed that ELF4 sequesters GI into nuclear bodies, thereby preventing GI to associate with the *CO* promoter (Kim et al., 2013b). It would be interesting to know the nature of the GI nuclear bodies and the components there in, using biochemical approach followed by mass spectrometric analysis.

**FIGURE 6:**
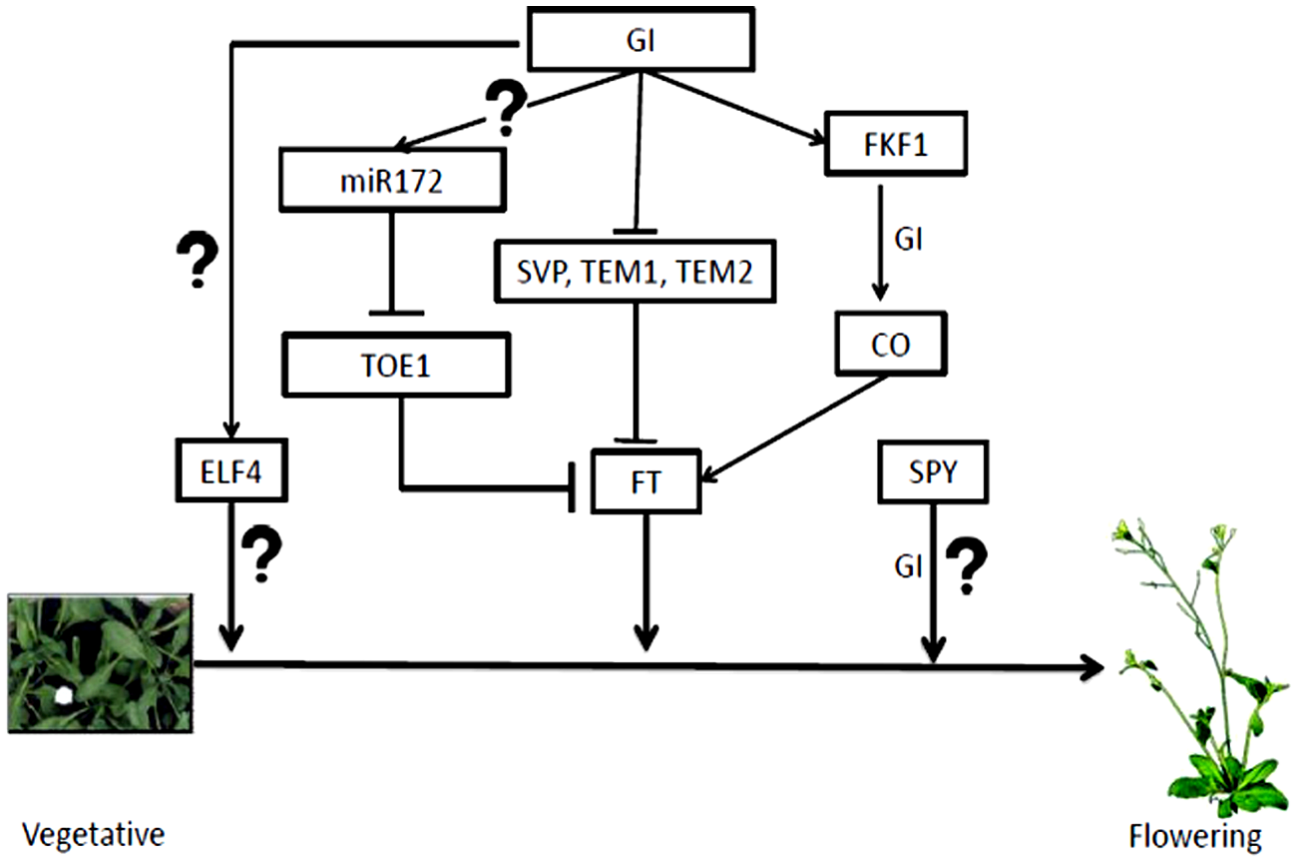
**Flowering time regulation by GI.** GI regulates flowering time through many pathways mostly up-regulating *FT.* The mechanism of flowering time control by GI along with ELF4 and SPY is unknown. The *miR172* processed by GI inhibits TOE1/2 that up-regulates *FT* transcription. GI also degrades inhibitors of *FT* transcription like SVP, TEM1 and 2. GI-FKF1 complex tunes *CO* transcription, which in turn controls *FT* accumulation.

GIGANTEA interacts with N-terminal tetracopeptide domains of SPINDLY (SPY), a plant O-linked β-*N*-acetylglucosamine transferase, and antagonizes its activity, thereby, promoting flowering (Tseng et al., 2004). Acetylglucosamine transferases have role in the addition of acetylglucosamine residues to proteins, which often competes with phosphorylation. This suggests that sugar modification may function as an important event in flowering time regulation. The known pathways through which GI regulates flowering are summarized in the **Figure 6**.

### PLEOTROPIC FUNCTIONS OF GIGANTEA

Besides flowering time, circadian clock, and light signaling regulation, GI has been implicated in other processes such as, sucrose signaling (Dalchau et al., 2011), starch accumulation (Eimert et al., 1995), and stress tolerance (Kurepa et al., 1998a; Fowler and Thomashow, 2002; Kim et al., 2013a; Riboni et al., 2013). The control of cotyledon movement, transpiration, and hypocotyl elongation responses have been shown to be attributed to the concerted activity of SPY and GI (Sothern et al., 2002; Tseng et al., 2004). The precise nature of this interaction is still unclear. However, GI functions antagonistically to SPY. The interaction of GI with SPY and ELF4 independently regulates hypocotyl length, where mutation in ELF4 and SPY are epistatic to *gi-2*.

GIGANTEA has been demonstrated to play a role between sucrose signaling and the circadian clock while grown in DD (Dalchau et al., 2011). Plants entrained in LD when shifted to DD, maintained the rhythmic *GI* expression exclusively in the presence of sucrose suggesting light independent control of GI rhythmicity. Although contradictory evidence on role of sucrose on *GI* expression has been reported, sucrose seems to affect the *GI* expression through *SENSITIVE TO FREEZING6* (*SFR6*) locus (Knight et al., 2008; Usadel et al., 2008). More precise experiments are required to unravel this mechanism. In the leaves of *Arabidopsis*, starch accumulation is elevated in the *gi* mutants (Eimert et al., 1995). On the contrary, presence of multiple copies of *GI* led to starch accumulation in the progeny of a cross between *A. thaliana* and *A. arenosa*, suggesting the antagonistic role of GI in these plants (Ni et al., 2009).

The *gi-3* mutants showed higher tolerance capacity to redox cycling agent, paraquat, and H_2_O_2_ (Kurepa et al., 1998a). Tolerance against paraquat is counteracted by the exogenously applied polyamines such as spermidine, spermine, and putrescine (Kurepa et al., 1998b). Paraquat treatment upregulated endogenous levels of putrescine in *gi-3* and WT. Since exogenous application of polyamines is effective for the resistance, the mechanism of the transporters during this stress needs attention. Oxidative stress due to herbicide imazethapyr has been shown to increase *GI* abundance and cause earlier flowering by ∼4 days (Qian et al., 2014). The mechanism behind higher tolerance to oxidative stress mediated by GI is still unclear.

Kurepa et al. (1998a) showed that *gi* mutants, *gi-3*, *gi-4*, *gi-5,* and *gi-6,* have more chlorophyll accumulation in comparison to WT in presence of paraquat. Even treatment with nitric oxide (NO) reduces the *GI* mRNA abundance and increases the chlorophyll content (He et al., 2004). In both the cases above, lower abundance of functional GI can be correlated to higher accumulation of chlorophyll. The role of GI in regulating the chlorophyll content needs to be studied in mutants and over-expressors of GI. Chlorophyll accumulation in allotetraploid, obtained by a cross between *A. thaliana* and *A. arenosa,* is higher than the WT individuals (Ni et al., 2009). The starch and chlorophyll accumulation in allotetraploids is exactly opposite in comparison to that seen in *A. thaliana*. The reverse trend might be due to post-transcriptional silencing posed by the presence of multiple homologous sequences of *GI* transcript, essentially a co-suppression phenomenon.

Dynamin, a GTPase having role in vesicle recycling during endocytosis, was found to interact with TAP tagged GI in rice (Abe et al., 2008). Although mutation in *dynamin* gene did not have any effect on the flowering time, it showed aerial rosette phenotype in *Arabidopsis*. In *Arabidopsis*, GI has been found to be involved in setting of fruits (Brock et al., 2007). No significant association of the GI haplogroup was detected with days to flower, petiole length, and inflorescence height. A significant association was observed between one haplogroup with fruit set, producing 14% more fruit than other haplogroups. Such studies in the crop plants could help in increasing the yield.

*GIGANTEA* mRNA levels increases about five- to eightfold in the cold treated *Arabidopsis* plants suggesting that *GI* is a cold-responsive gene (Fowler and Thomashow, 2002). The flowering time of *gi* mutants was further delayed when exposed to low temperature compared to WT (Cao et al., 2005). C-repeat Binding proteins (CBFs) have been known to regulate various genes responsive to cold and are implicated in cold stress tolerance. On the contrary, Cao et al. (2005), it was revealed that GI regulates cold acclimation through CBF-independent pathway. The ability to tolerate and acclimatize toward cold is reduced in *gi* mutants suggesting the protective role of GI in cold tolerance.

Recently, the role of GI under salt stress was documented (Kim et al., 2013a). Although, salt stress did not affect the *GI* expression, it affected the GI protein stability in *pGI::GI-HA* transgenics (Cao et al., 2005; Kim et al., 2013a). It seems plausible that there is a mechanism at the post-translational level that regulates GI abundance. GI also regulated the activities of the proteins involved in the salt stress tolerance. It interacts with Salt Overly Sensitive 2 (SOS2) directly and inhibits the activity of SOS1, a Na^+^/H^+^ anti-porter. Therefore, GI is a negative regulator of salt tolerance and is degraded during salt stress. According to a recent model, plants under salt stress would flower later than when grown in normal growth conditions reasoned for the degradation of GI (Park et al., 2013).

In *At* and other plants, the tolerance to higher salinity, enhanced cold, and sustained drought were manifested by the increase of sub-cellular level of abscisic acid (ABA). Recent reports indicated that GI has role in ABA-dependent drought escape tolerance. It suggests that the GI regulation of salt and cold stress tolerance could very likely be ABA-mediated (Riboni et al., 2013). Drought stress up-regulates *GI* transcription and in turn, increases the abundance of *miR172E* variant (Han et al., 2013). *WRKY DNA binding protein 44* (*WRKY44*) was found to be suppressed by GI in drought stress and interact with TOE1. *GI-miR172-WRKY44* were proposed to be in the same pathway possibly associated with drought stress tolerance. On the same line of thinking, the light dependent GI-mediated stomatal opening response could be ABA mediated (Ando et al., 2013). GI also has a role in wall in-growth deposition in phloem parenchyma transfer cells in *A. thaliana* in response to high light and cold stress (Edwards et al., 2010; Chinnappa et al., 2013).

## ROLE OF GIGANTEA HOMOLOGS

GIGANTEA homologs in prokaryotes, fungi, mosses, or animals have not been reported as yet (Holm et al., 2010). GI homolog has been shown to be absent in the green unicellular alga *Ostreococcus tauri* (Corellou et al., 2009). Evolution of GI has been correlated with the evolution of higher plants from liverwort onward, although being absent in mosses. The evolution of GI can be proposed to have taken place alongside the origin of land plants. The role of GI in light signaling, circadian clock control, and flowering time regulation seems to be conserved across the plant kingdom, as inferred from the various studies to understand the role of GI homologs in *Arabidopsis*. GI homologs from the non-flowering and flowering plants have been summarized below.

The GI-FKF1 interaction and function has been recently shown to be conserved in the liverwort *Marchantia polymorpha* (Kubota et al., 2014). The LOV domain of FKF1, which has been found to be required for the interaction with GI, contains a conserved cysteine residue in AtGI and MpGI important for its blue light dependent functions (Sawa et al., 2007). GI-FKF1 module has been proposed to be important for the transition of plants from water to land and the evolution of vascular system. The *Marchantia polymorpha* ortholog of *GI, MpGI*, has been shown to partially rescue the late flowering phenotype of *Arabidopsis gi* mutant suggesting the functional conservation of GI across the plant kingdom. The FKF1 homologs have been shown to be present in *A. thaliana* (AtFKF1, AtZTL, and AtLKP2), *Oryza sativa* (OsFKF1, OsZTL1, and OsZTL2), *Glycine max* (GmFKF1, GmZTL1, and GmZTL2), *Triticum aestivum* (TaFKF1 and TaZTL), *Allium cepa* (AcFKF1 and AcZTL), *Mesembryanthemum crystallinum* (McFKF1 and McZTL), and *Selaginella moellendorffii* (SmFKF; Kubota et al., 2014). The GI counterparts in the above mentioned species are also conserved. This shows that GI-FKF1 module has been conserved since the primitive time and thus might be have been important in shaping the development of higher plant. This light perceiving module needs to be studied in detail to understand the evolution of various functions and residues along with putative domains required to carry out these functions in plants. The conserved interaction of GI with FKF1 has been shown to be conserved in soybean.

### GYMNOSPERM

#### Norway Spruce (*Picea abies*)

GIGANTEA ortholog of *Picea abies,* PaGI and AtGI share 58% identity and 72% similarity. Natural variations in *GI* have been correlated to clinal variations in the different populations of close relative of the Scandinavian Norway spruce (Chen et al., 2014). Over-expression of *PaGI* in WT *Arabidopsis* did not show any phenotypical changes (Karlgren et al., 2013). However, when PaGI was over-expressed in *gi-2* mutant, it partially rescued the late flowering phenotype and flowered at the same time as WT plants suggesting that PaGI and AtGI are functionally conserved to large extent. The strength of the over-expression has neither been verified at the gene expression level nor the protein accumulation level and therefore needs to be confirmed.

### ANGIOSPERMS (MONOCOTS)

#### Barley (*Hordeum vulgare*)

GIGANTEA homolog in Barley was identified using BLAST searches and later confirmed by Southern hybridization analyses. Only one homolog was detected in barley. Barley *GI* (*HvGI*) has ∼94 and ∼79% similarity with *OsGI* and *AtGI,* respectively (Dunford et al., 2005). Barley, being a long-day plant, its *GI* expression followed the pattern documented for *AtGI*. Characteristically, in SDs, the peak of expression was noticed about 6–9 h after dawn whereas, in LDs, the peak is shifted to 15 h after dawn (Dunford et al., 2005). The mutation in *HvELF3* (*mat-a.8*), a 4 bp deletion causing frame shift and premature stop codon, was found in the barley cultivar Mari (Zakhrabekova et al., 2012). This mutation led to the up-regulation of *HvGI* transcription and was found to be the reason for early flowering phenotype in this cultivar. Although, post-translational interaction between AtELF3 and AtGI is known, no evidence is there in *Arabidopsis* suggesting the transcriptional regulation of *GI* by ELF3.

#### Duckweed (*Lemna gibba*)

The *AtGI* homolog of *L. gibba*, *LgGIH1,* a LD plant, plays a pivotal role in its circadian clock control, since the *LgGIH1* knockdown resulted in the arrhythmic gene expression phenotype in plants (Serikawa et al., 2008). Earlier reports suggested that *AtGI* and *LgGIH1* followed similar expression pattern in both LD and LL conditions (Miwa et al., 2006). The function of GI and ELF3 homologs are shown to be conserved between *Arabidopsis* and *L. gibba*.

#### Maize (*Zea mays*)

Maize is a SD plant, which has two diurnally regulated *GI* homologs called *gigantea of Z. mays 1a and 1b (gi1 and 2)* due to tetraploidy events and genome evolution expressed in leaves (Gaut and Doebley, 1997; Swigonová et al., 2004; Miller et al., 2008; Hayes et al., 2010; Khan et al., 2010; Schnable et al., 2011). Among the two homologs, the *gigantea1* transcript was highly expressed. Mutation in *gi1* caused early flowering in LD but had lesser effects in SD. The *gi1* mutation also increases plant height and alters the timing of the vegetative phase (Bendix et al., 2013). The early flowering phenotype of *gi1* mutant was due to the conserved pathway involving the up-regulation of *CO*-like flowering regulatory gene called *CO of Z. mays1* (*conz1*) and *FT*-like floral activator gene named *Z. mays centroradialis8* (*zcn8*).

#### Purple False Brome (*Brachypodium distachyon*)

GIGANTEA ortholog of *B. distachyon (BdGI)* is rhythmically regulated by the circadian clock and up-regulated by both cold and dark (Hong et al., 2010). *BdGI* was identified by BLAST search followed by Southern hybridization analysis. The *BdGI* transcript level was found to be oscillating in both SD and LD conditions, like *AtGI*. While the lowest transcript level in both SD and LD was at ZT 0, the peak in SD was at ZT 8 and in LD was at ZT 12. BdGI shares 65% identity with AtGI. BdGI, like AtGI, is a nuclear localizing protein and interacts with COP1 and ZTL proteins as evident from the yeast two-hybrid assays. BdGI complements the late flowering phenotype of *Arabidopsis gi-2* mutant suggesting the conserved function of GI in monocots and dicots. While PhyC does not show a pronounced effect in the LD model *Arabidopsis*, it causes late flowering in this temperate grass (Woods et al., 2014). In *phyC* mutants, *GI* expression is almost undetectable. The low *GI* expression could explain the lower abundance of the homologs of *CO* and *FT.* The delayed flowering phenotype suggests that the photoperiodic flowering pathway through GI is conserved in grasses as in *Arabidopsis*.

#### Rice (*Oryza sativa*)

Rice and *Arabidopsis GI* share 67% similarity and the NLS are quite conserved between *OsGI* and *AtGI* (Hayama et al., 2002). *GI* expression pattern was similar in both rice and *At* (Hayama et al., 2002) and similarly, OsGI acts as a positive regulator of *Hd1* (*CO* homolog of rice; Hayama et al., 2003). It controls the rhythm of nearly 27000 genes in rice (Izawa et al., 2011). When *gi* mutants are grown in field conditions, sucrose, and starch content increases, chlorophyll content decreases, stomatal conductance increases, panicle, and spikelet number increases and fertility was reduced. OsGI was shown to be involved in ETR2 (ethylene receptor)-dependent late flowering phenotype and starch accumulation thus, regulating the developmental transition based on the availability of energy (Wuriyanghan et al., 2009).

#### Tulip (*Liriodendron tulipifera*)

GIGANTEA ortholog was shown to be closer to eudicot GI sequence than the monocot sequences (Liang et al., 2010).

#### Wheat (*Triticum aestivum* L.)

Wheat is a LD plant and has been shown to have an ortholog of *AtGI*, referred as *TaGI1* (Zhao et al., 2005). *TaGI1* has ∼81 and 63% identity with *OsGI* and *AtGI,* respectively. The *TaGI1* follow rhythmic pattern of expression similar to that of *Arabidopsis* and over-expression of *TaGI1* complements late flowering phenotype of *gi-2* mutant *Arabidopsis*. *TaGI* was also associated with “earliness phenotype” of wheat which helps in its adaptation and increase in yield in varied environmental conditions (Rousset et al., 2011).

### ANGIOSPERMS (DICOTS)

#### Common Ice Plant (*Mesembryanthemum crystallinum*)

A crassulacean acid metabolism plant, *Mesembryanthemum crystallinum*, also showed a rhythmic expression of the orthologs of *GI, McGI* (Boxall et al., 2005). The ortholog was identified using BLAST search and later isolated and sequenced. *McGI* expression peaks at ZT 9 similar to *AtGI*.

#### Morning Glory (*Pharbitis nil*)

PnGI protein shares 70 and 67% identity with AtGI and OsGI protein, respectively (Higuchi et al., 2011). *PnGI* mRNA is also circadian regulated like the other GI orthologs. Over-expression of *PnGI* led to altered period length affecting the expression pattern of downstream genes. *Pharbitis nil* is a SD plant, and like OsGI, PnGI inhibits the expression of *PnFT* (*FT* homolog of morning glory).

#### Pea (*Pisum sativum*)

*LATE BLOOMER 1* (LATE1) is the *AtGI* ortholog in pea, a LD plant, and follows a rhythmic pattern of expression as seen in *Arabidopsis* (Hecht et al., 2007). LATE 1 was shown to be regulating the pea homologs of *Arabidopsis* circadian clock genes. Apart from its role in flowering time and circadian clock regulation, LATE1 has been implicated in Phy-B dependent seed de-etiolation in red light. LATE1 was found to regulate circadian clock gene expression in constant light and dark (Liew et al., 2009). In LD and SD, LATE1 was shown to control a mobile signal that regulates the flowering time.

#### Radish (*Raphanus sativa*)

In another instance, expression of antisense *AtGI* gene, under the constitutive 35S promoter, led to delayed bolting in LDs, proving that GI has an important role in photoperiodic flowering time control in this plant (Curtis et al., 2002). The bolting and flowering time was delayed by 17 and 18 days, respectively, with respect to WT plants.

#### Soybean (*Glycine max*)

*Glycine max,* a SD plant, has two GI orthologs – GmGIa and GmGIb (Watanabe et al., 2011). Both the *GmGI* sequences have nearly 70–91% identity to eudicot and monocot genes. Like OsGI, GmGI regulated GmFT paralogs. GmGI has been shown to have role in soybean seed maturity. *GmGI* loss of function leads to early flowering as in the model SD rice plant. Interestingly, a recent study in soybean suggested that there are three *AtGI* homologs in the soybean genome unlike previously suggested two orthologs GmGIa and GmGIb (Li et al., 2013). The third form is a result of alternative splice form of *GmGIa,* resulting in *GmGI*α and *GmGI*β. The GI orthologs were diurnally regulated and differentially expressed in different tissues adding up to a more complex regulation. GmGI proteins have the conserved NLS and localize to nucleus. GmGI proteins have been shown to interact with orthologs of FKF1 in soybean suggesting that function most likely is conserved.

#### Tomato (*Solanum lycopersicum*)

Tomato is a day neutral plant. GI was shown to be up-regulated and inhibit tomato seed germination thereby promoting seed dormancy under FR condition in the presence of functional PhyA (Auge et al., 2009). On the contrary, in *Arabidopsis*, loss of function of GI led to elevated dormancy (Penfield and Hall, 2009). In other members of the Solanaceae such as potato and tobacco, photoperiodic control of GI was also shown to be operational (Rutitzky et al., 2009).

The conserved diurnal regulation of *GI* in different plants described above suggests the prevalence of an important transcriptional machinery as well as the *GI* promoter. The availability of GI antiserum would help to understand the regulation of *GI* in these crop plants. The localization and the stability of GI in most of these plants are still to be addressed. While few of the interaction with proteins such as orthologs of ELF3, COP1, ZTL, and FKF1 are shown to be conserved, the function of these complexes needs to be disclosed in various species.

## CONCLUSION AND PERSPECTIVE

GIGANTEA seems to be a very important plant protein involved in various processes, from developmental regulation to metabolic flux. Despite its pivotal roles, it is surprising that *GI* null mutants are not lethal. Being a large protein, it might satisfy to function in several pathways summarized, yet to be fully understood. It would be a great challenge to understand and connect the functional roles of GI at different developmental stages. Although GI is a multifunctional protein, the role of its various functional domains are still in darkness. A functional antiserum against a conserved domain of GI that would detect the endogenous level of protein across species and in multiple mutational background would be very useful. The lack of such an antiserum possesses a serious bottleneck delaying the understanding of its abundance, regulation at the protein level and regulatory functions like the GI-FKF1 module across the plant kingdom. Despite this problem, several elegant experiments have been published where researchers have attempted to understand its role using transgenic plants expressing tagged versions of GI. Although time-consuming, these are the impressive feats that place GI mechanistically in a network of photoperiod control pathway.

The role of GI in flowering time regulation, circadian clock control, and light signaling is still being pursued. But less-known functions such as sucrose signaling, chlorophyll accumulation, oxidative stress resistance demand more attention. More functions of GI are beginning to be documented. Recently, the emerging role of GI in salt tolerance has been demonstrated, which indicates that we are still not saturated in understanding the various functions GI. It would be interesting to understand how GI regulates so many functions before going into the complex cross talk between them it can fine tune. The lower plant moss *Physcomitrella patens* does not have a GI ortholog but still carries out most of the developmental aspects except flowering. It is very interesting to note that they do have CO-like genes, therefore the evolution of GI function is still an interesting area and demands further attention (Zobell et al., 2005).

## Conflict of Interest Statement

The authors declare that the research was conducted in the absence of any commercial or financial relationships that could be construed as a potential conflict of interest.
